# Genome-wide identification and analysis of the cotton ALDH gene family

**DOI:** 10.1186/s12864-024-10388-x

**Published:** 2024-05-24

**Authors:** Haijing Gu, Zongjin Pan, Mengxue Jia, Hui Fang, Junyi Li, Yingxiao Qi, Yixuan Yang, Wenxiang Feng, Xin Gao, Allah Ditta, Muhammad K.R. Khan, Wei Wang, Yunying Cao, Baohua Wang

**Affiliations:** 1 School of Life Sciences, Nantong University, Nantong, Jiangsu 226019 China; 2 Jiangsu Coastal Area Institute of Agricultural Sciences/Jiangsu Collaborative Innovation Center for Modern Crop Production, Yancheng, Jiangsu 224002 China; 3 Nantong Middle School, Nantong, Jiangsu 226001 China; 4 Plant Breeding and Genetics Division, Nuclear Institute for Agriculture and Biology, Faisalabad, 38000 Pakistan

**Keywords:** Cotton, ALDH, Salt stress, Gene family analysis, Virus-induced gene silencing

## Abstract

**Background:**

Aldehyde dehydrogenases (ALDHs) are a family of enzymes that catalyze the oxidation of aldehyde molecules into the corresponding carboxylic acid, regulate the balance of aldehydes and protect plants from the poisoning caused by excessive accumulation of aldehydes; however, this gene family has rarely been studied in cotton.

**Results:**

In the present study, genome-wide identification was performed, and a total of 114 ALDH family members were found in three cotton species, *Gossypium hirsutum*, *Gossypium arboreum* and *Gossypium raimondii*. The ALDH genes were divided into six subgroups by evolutionary analysis. ALDH genes in the same subgroup showed similar gene structures and conserved motifs, but some genes showed significant differences, which may result in functional differences. Chromosomal location analysis and selective pressure analysis revealed that the ALDH gene family had experienced many fragment duplication events. *Cis*-acting element analysis revealed that this gene family may be involved in the response to various biotic and abiotic stresses. The RT‒qPCR results showed that the expression levels of some members of this gene family were significantly increased under salt stress conditions. *Gohir.A11G040800* and *Gohir.D06G046200* were subjected to virus-induced gene silencing (VIGS) experiments, and the sensitivity of the silenced plants to salt stress was significantly greater than that of the negative control plants, suggesting that *Gohir.A11G040800* and *Gohir.D06G046200* may be involved in the response of cotton to salt stress.

**Conclusions:**

In total, 114 ALDH genes were identified in three Gossypium species by a series of bioinformatics analysis. Gene silencing of the ALDH genes of *G. hirsutum* revealed that ALDH plays an important role in the response of cotton to salt stress.

**Supplementary Information:**

The online version contains supplementary material available at 10.1186/s12864-024-10388-x.

## Background

Aldehyde dehydrogenases (ALDHs) are a family of enzymes that catalyze the oxidation of aldehydes to their corresponding carboxylic acids [1]. Aldehydes are a class of highly active toxic substances that are mainly produced by membrane lipid peroxidation, amino acid oxidation and protein glycosylation in organisms. Aldehyde molecules are common intermediates in various catabolic and biosynthetic pathways. Their production is the result of responses to biotic and abiotic stresses. Although aldehydes are indispensable for the growth and development of organisms, excessive amounts of aldehydes can interfere with metabolism and become toxic substances. Therefore, organisms must regulate cells to maintain a balanced level [2, 3]. Aldehyde dehydrogenase is usually associated with the detoxification of aldehydes [4], which helps prevent the excessive generation of oxygen free radicals, reduces the oxidative stress in cells, and maintains the stability of intracellular biomolecules, which is critical for cell health and normal organism function.

ALDH exists in almost all organisms [5] and plays an important role in plants. Zhao et al. found that the ALDH gene plays a crucial role in protecting plants from high temperature damage by generating ALDH-overexpressing plants in *Arabidopsis thaliana* [6]. Cao et al. reported that the introduction of ALDH protein into soybeans can increase the tolerance of transgenic plants to saline-alkali stress by maintaining cell wall structure and metabolite transport [7]. In addition, ALDH has been found to participate in plant stress resistance in tobacco [8], maize [9] and millet [10]. The ALDH gene family has also been studied in cotton. Guo et al. reported that the ALDH gene family responds to high salt and high drought in *Gossypium arboreum* and *Gossypium hirsutum* [11], and He et al. reported that the ALDH gene family plays a role in coping with drought and flood stress in *Gossypium raimondii* [12]. Dong et al. analyzed the expression profiles of the ALDH gene family in four representative tissues of *Gossypium hirsutum*: roots, stems, cotyledons, and leaves. They found that ALDH genes were expressed in all four representative tissues of *G. hirsutum*. Additionally, they analyzed the changes in the expression levels of ALDH genes in these four representative tissues under salt stress. Despite roots being the tissues directly exposed to salt stress, they found that under severe salt stress (200 mmol), the expression levels of most ALDH genes were upregulated in leaves [13]. All three authors validated the expression levels of ALDH genes under abiotic stress conditions using RT-qPCR. Moreover, similar results were also obtained via bioinformatics analysis of the ALDH family. For instance, Guo and Dong et al. both discovered the presence of low-temperature responsive LTR elements in the promoter regions of *Gossypium hirsutum*. Additionally, they conducted analyses on the intron-exon structure of the ALDH gene family and found it to be conserved during evolution. Yang et al. isolated an ALDH gene from a drought-tolerant moss, and its overexpression in cotton confirmed that the ALDH family played a role in cotton drought tolerance [14].

Cotton is a pioneer crop in saline-alkali land and is also an important fiber and oil crop [15]. High soil salinity strongly affects the growth and production of cotton. Compared with other stages, the germination and emergence stages are more susceptible to salt stress [16], so it is particularly important to explore the salt stress tolerance mechanism at the seedling stage. To compensate for the lack of information on the ALDH gene family in cotton research, this study downloaded the latest genomic data of *Gossypium hirsutum*, *Gossypium arboreum* and *Gossypium raimondii* from the database and revealed their evolutionary relationships, *cis*-acting elements, gene structures, conserved motifs, chromosome locations and other information through a series of bioinformatics methods. Virus-induced gene silencing (VIGS) was used to investigate the response of this gene family to salt stress in upland cotton at the seedling stage. This study provides a theoretical reference for revealing the genetic evolution, growth and regulatory mechanisms of the cotton ALDH gene family in response to salt stress.

## Results

### Identification and phylogenetic relationship analysis of ALDH gene families in cotton

SMART and Pfam tools were used to verify the ALDH domain in the ALDH protein sequence. Overall, 57 ALDH proteins were identified in *G. hirsutum*, 30 in *G. arboreum*, and 27 in *G. raimondii* (Supplementary Table 1, Table S1). All of these proteins contain at least one conserved ALDH domain.

**Fig. 1:**
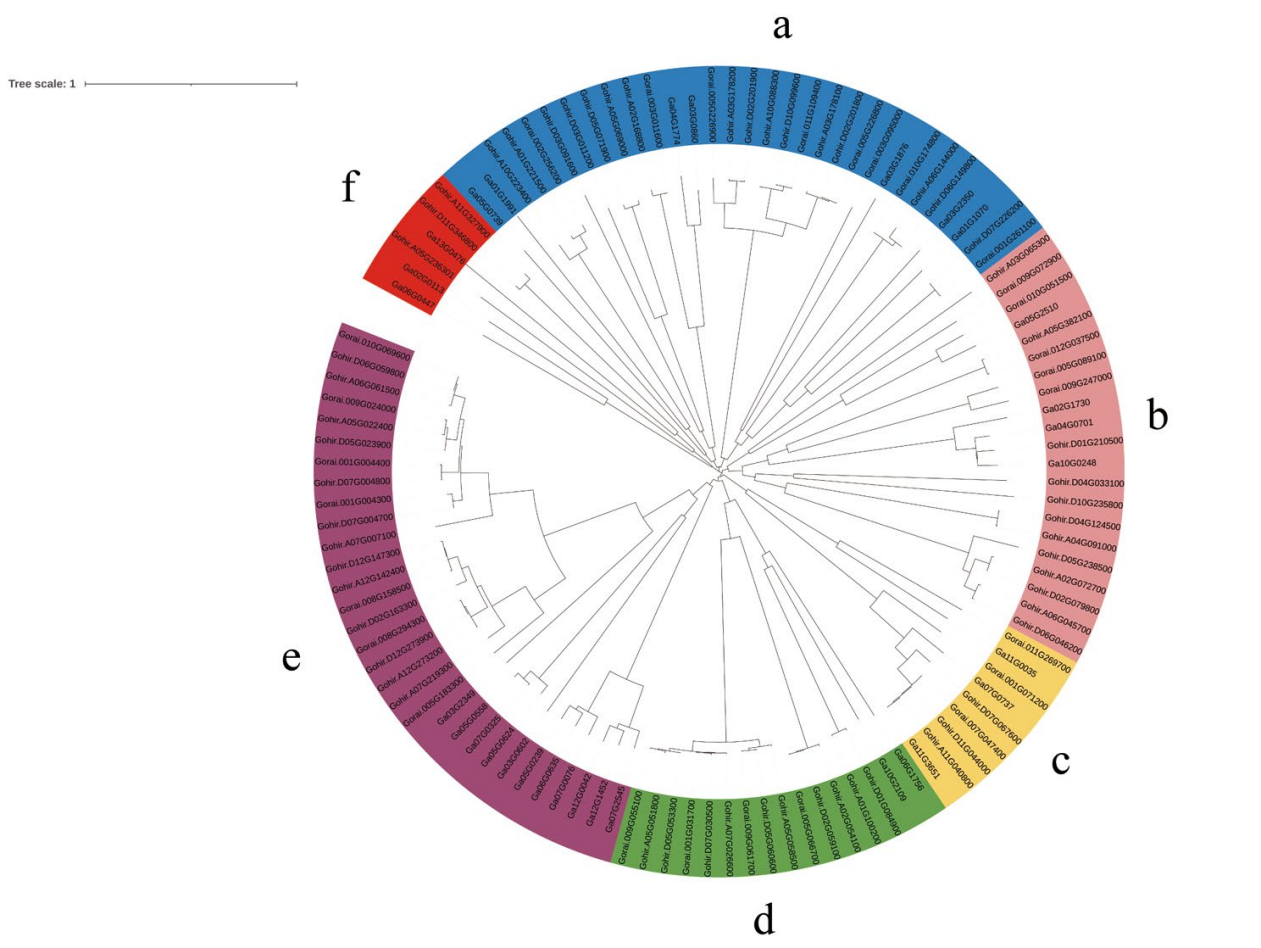
Phylogenetic tree of the ALDH gene family in *Gossypium hirsutum*, *Gossypium arboreum*, and *Gossypium raimondii*. Gohir: *Gossypium hirsutum*, Gorai: *Gossypium raimondii*. Ga: *Gossypium arboreum*. cluster A on blue background, cluster B on light pink background, cluster C on yellow background, cluster D on green background, cluster E on deep purple background and cluster F on red background

We performed multiple sequence comparisons of these 114 genes to construct phylogenetic trees to analyze the evolutionary relationships between the genes. As shown in Fig. 1, these 114 genes can be divided into 6 subgroups, each consisting of 6, 31, 21, 9, 16 and 31 genes, respectively, and we speculate that members of the same clade have more similar evolutionary relationships and more similar functions.

### Evolutionary selection pressure analysis of the ALDH gene family

*G. hirsutum* is a heterotetraploid species formed by hybridization of diploid A-genome (*G. arboretum*) and D-genome (*G. raimondii*) cotton. Calculator 2.0 was used to calculate the synonymous substitution rates (Ka) and nonsynonymous substitution rates (Ks) of nucleotides for ALDH genes. The Ka/Ks ratio was further calculated to analyze the selection of ALDH gene families during phylogeny. During the process of evolution, amino acids may undergo some corresponding changes due to nonsynonymous replacement of genes (Ka), which can cause changes in protein structure and function. Ka can reflect the functional changes in protein-coding genes or the diversity of protein sequences. Ks represents the rate of synonymous substitution (no amino acid changes but codon changes) that occurs in a gene or protein sequence. Ks measures changes in nucleotides between different versions of a gene or protein sequence that do not result in changes in amino acids. The Ka/Ks ratio of genes was used to analyze the species selection pressure. During the development and evolution of most ALDH genes in *G. hirsutum*, the synonymous substitution rate of bases was significantly greater than the nonsynonymous substitution rate, so it was not affected by natural selection. The results indicated that these genes have undergone neutral selection during evolution. Moreover, some genes had Ka/Ks ratios greater than 1, e.g., *Gohir. A05G069000 and Gohir. D05G071900*, *Gohir. D11G346800* and *Gohir. A04G091000*, *Gohir. D11G346800* and *Gohir. D01G210500*, *Gohir. D11G346800* and *Gohir. A01G221500* (Supplementary Table 2, Table S2), suggesting that these genes may have been subject to positive selection and may have undergone functional changes.

### Chromosome localization and fragment repeat analysis of ALDHs

**Fig. 2:**
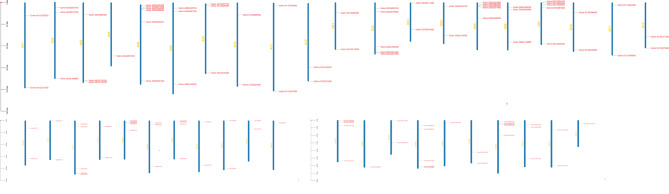
Chromosomal distribution of the ALDH gene family in *Gossypium hirsutum*, *Gossypium arboreum*, and *Gossypium raimondii*. Gohir: *Gossypium hirsutum*, Gorai: *Gossypium raimondii*. Ga: *Gossypium arboreum*. The blue represents chromosomes. Yellow text on the left side of the chromosome denotes chromosome numbers, while gene IDs are on the right side

TBtools software was used to map the distribution of ALDHs on chromosomes of the three cotton species. Among the 57 ALDH gene family members in *G. hirsutum*, 29 members were distributed on 11 At chromosomes, namely, Chromosomes A01, A02, A03, A04, A05, A06, A07, A10, A11, A12 and A13. The other 28 members are distributed on the 10 Dt chromosomes, including Chromosomes D01, D02, D03, D04, D05, D06, D07, D10, D11 and D12. The 30 ALDH gene family members in *G. arboretum* are distributed on 10 A genome chromosomes, namely, Chromosomes A01, A02, A03, A04, A05, A06, A07, A10, A11, A12, and A13. In *G. raimondii*, 27 ALDH gene family members are distributed on 10 D genome chromosomes, namely, Chromosomes D01, D02, D03, D05, D07, D08, D09, D10, D11 and D12 (Fig. 2). The diploid and tetraploid cotton species have an uneven distribution of chromosomes in the D subgenome, suggesting that these genes may have been produced after the cotton polyploid event. Two genes from Chromosomes D08 and D09 in *G. raimondii* were not found in *G. hirsutum*, suggesting that these two genes may have been eliminated during evolution.

### Analysis of gene structure and conserved motifs of the ALDH gene family

The exon/intron distribution of the ALDH gene was analyzed by using the GSDS tool. To better understand the evolutionary relationships among different members of the ALDH gene family, phylogenetic trees were constructed using ALDH protein sequences and the NJ method, and the exon‒intron structure and conserved motif of ALDH family members from different cotton species were compared. Despite differences in the exons and introns of ALDH genes, the more closely related genes in the evolutionary tree had more similar exon and intron arrangements across the three cotton species, suggesting that exon‒intron structure was associated with phylogenetic relationships between ALDH genes.

**Fig. 3:**
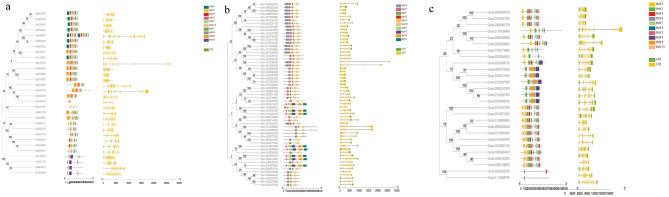
Phylogenetic tree, conserved motifs, and gene structure analysis of the ALDH family. **a**: *Gossypium arboreum*, **b**: *Gossypium hirsutum*, **c**: *Gossypium raimondii*. Note: The phylogenetic tree was constructed using MEGA 7 neighbor-joining (NJ) with 1000 bootstrap replicates. The conserved motifs in ALDH proteins were identified using MEME software. Gray lines indicate non-conserved sequences, and each motif is indicated by a colored box. The lengths of the motifs in each protein presented proportionally. The exon‒intron structure of the ALDH gene is based on evolutionary relationships. Yellow rectangles indicate exons and gray lines indicate introns

Conserved motifs are usually associated with the function of proteins. To reveal the characteristic motifs of ALDHs, MEME software was used to identify the conserved motifs in the ALDH protein (Fig. 3). A total of 10 conserved motifs were identified and named Motif 1 to Motif 10, and the number of conserved motifs in each ALDH protein ranged from 2 to 8. However, ALDH genes in the same branch had similar conserved motif distribution patterns. For example, in *G. hirsutum*, almost every protein contains Motif 3 and Motif 5; in *G. raimondii*, each protein contains Motif 1; and in *G. arboreum*, almost every protein contains Motif 2, suggesting that these motifs are highly conserved in these cotton species. Simultaneously, we submitted the predicted motif sequence information to the PFAM database for functional querying. The results revealed that these motifs are all associated with oxidoreductase activity and activity, and act on the aldehyde or oxo group of donors, or on NAD or NADP as acceptors.

### Analysis of *cis*-acting elements of the cotton ALDH gene family

**Fig. 4:**
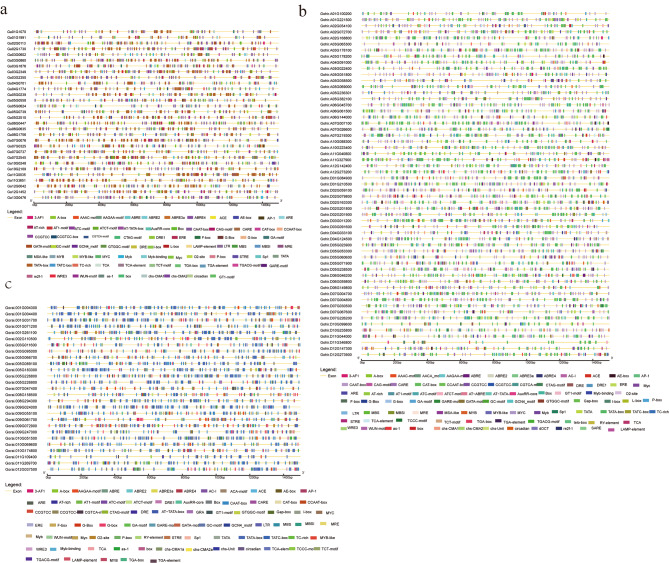
*Cis*-acting element analysis of the ALDH gene family. a: *Gossypium arboreum*, b: *Gossypium hirsutum*, c: *Gossypium raimondii*

To understand the potential function of the ALDH gene family, we extracted sequences 1400 bp upstream from the transcriptional start site from the cotton genome database and submitted these sequences to the PLANTCARE database to determine the distribution of their *cis*-acting elements. Many *cis*-acting elements involved in plant development and the response to stress, such as ABREs, AE-boxes, G-boxes, and CAAT-boxes, were detected in the promoter regions of ALDH genes (Fig. 4). The information and functions of these *cis*-regulatory elements are presented in Supplementary Table 3 (Table S3). These results suggest that ALDHs may play an important role in plant responses to biotic and abiotic stresses.

### Collinearity analysis of the cotton ALDH gene family

**Fig. 5:**
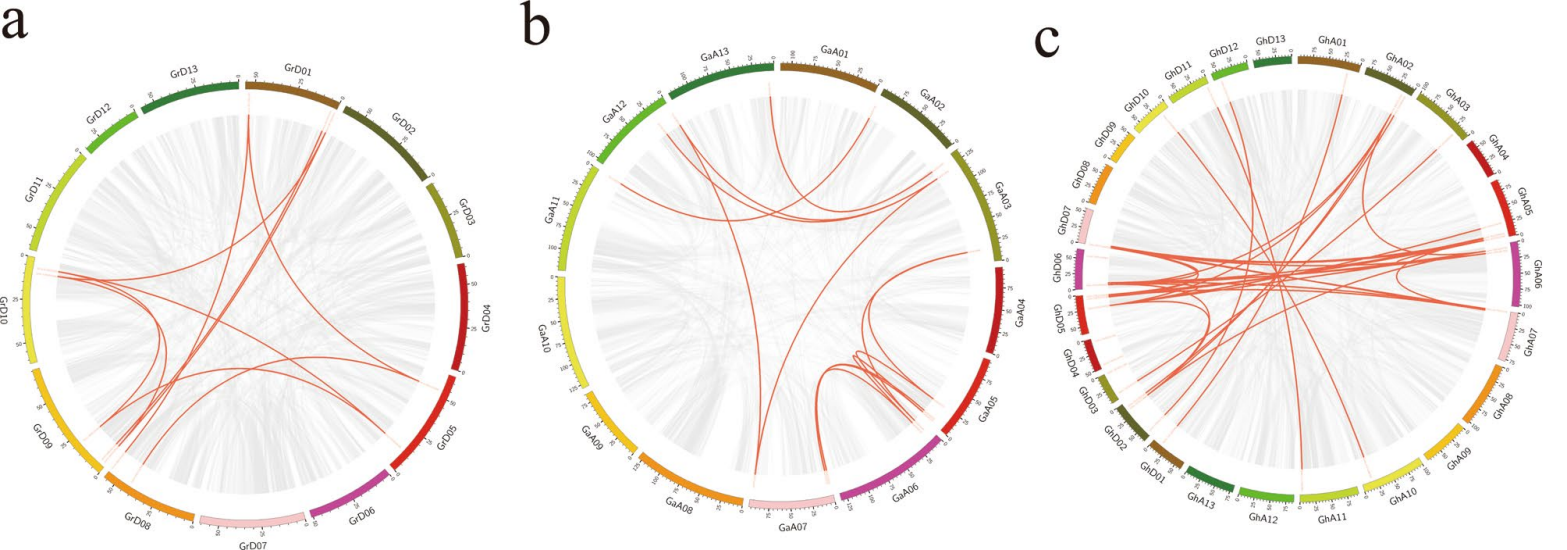
Interchromosomal relationships of ALDHs genes. Grey lines indicate all syntenic blocks in *Gossypium raimondii*, *Gossypium arboreum* and *Gossypium hirsutum* genome. Red lines indicate collinear blocks of ALDH genes in *Gossypium raimondii*, *Gossypium arboreum* and *Gossypium hirsutum*. a: *Gossypium raimondii*, b: *Gossypium arboreum*, c: *Gossypium hirsutum*

**Fig. 6:**
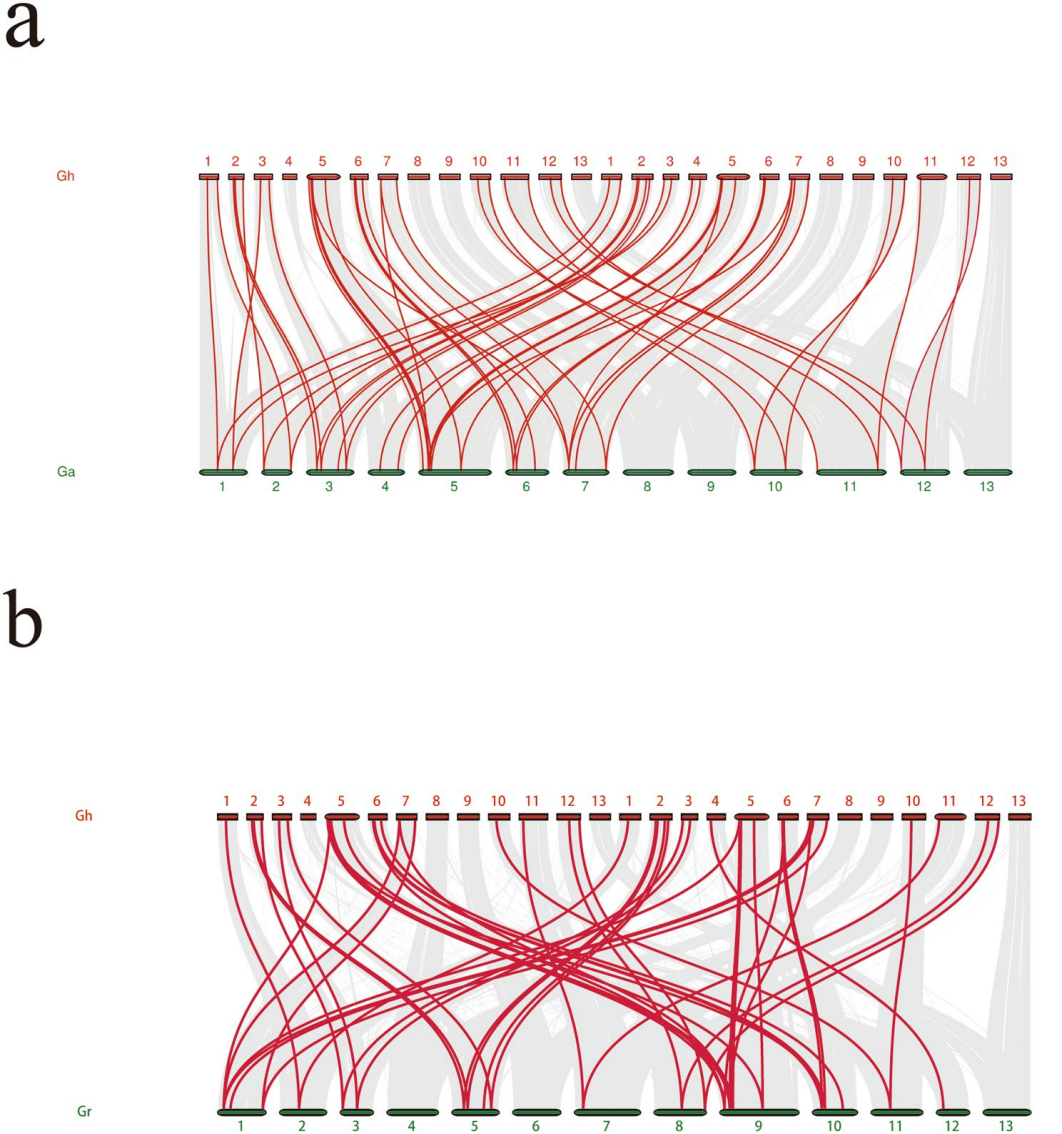
Syntenic analysis of ALDH genes between *Gossypium hirsutum* and its two ancestral species of it. a: Syntenic analysis of ALDH genes between *Gossypium hirsutum* and *Gossypium arboreum*. b: Syntenic analysis of ALDH genes between *Gossypium hirsutum* and *Gossypium raimondii*. Gray lines in the background indicate the collinear blocks within *Gossypium hirsutum* and other plant genomes, whereas red lines highlight syntenic ALDH gene pairs. Gh: *Gossypium hirsutum*, Ga: *Gossypium arboretum*, Gr: *Gossypium raimondii*

Collinearity analysis can reveal the origin and evolutionary history of gene families, including the process of replication, transposition and rearrangement of gene family members, as well as their functional changes and adaptive evolution during evolution. Therefore, we performed a collinearity analysis of ALDH gene families in three different cotton species using MCScanX and Circos software for mapping. Collinearity of the ALDH gene occured mainly between the D01 and D09 chromosomes in *G. raimondii* (Fig. 5a), between chromosomes A05 and A06 in *G. arboreum* (Fig. 5b), and between homologous chromosomes in tetraploid *G. hirsutum* (Fig. 5c). Since Raymond’s cotton and Asian cotton are two ancestors of upland cotton, we also analyzed the collinear relationships between upland cotton and these two ancestors. There was also a collinear relationship between the ALDH gene family in tetraploid *G. hirsutum* and the group A and D chromosomes of its ancestral species, *G. arboretum* and *G. raimondii* (Fig. 6a, b).

### RT‒qPCR analysis of the cotton ALDH gene family

**Fig. 7:**
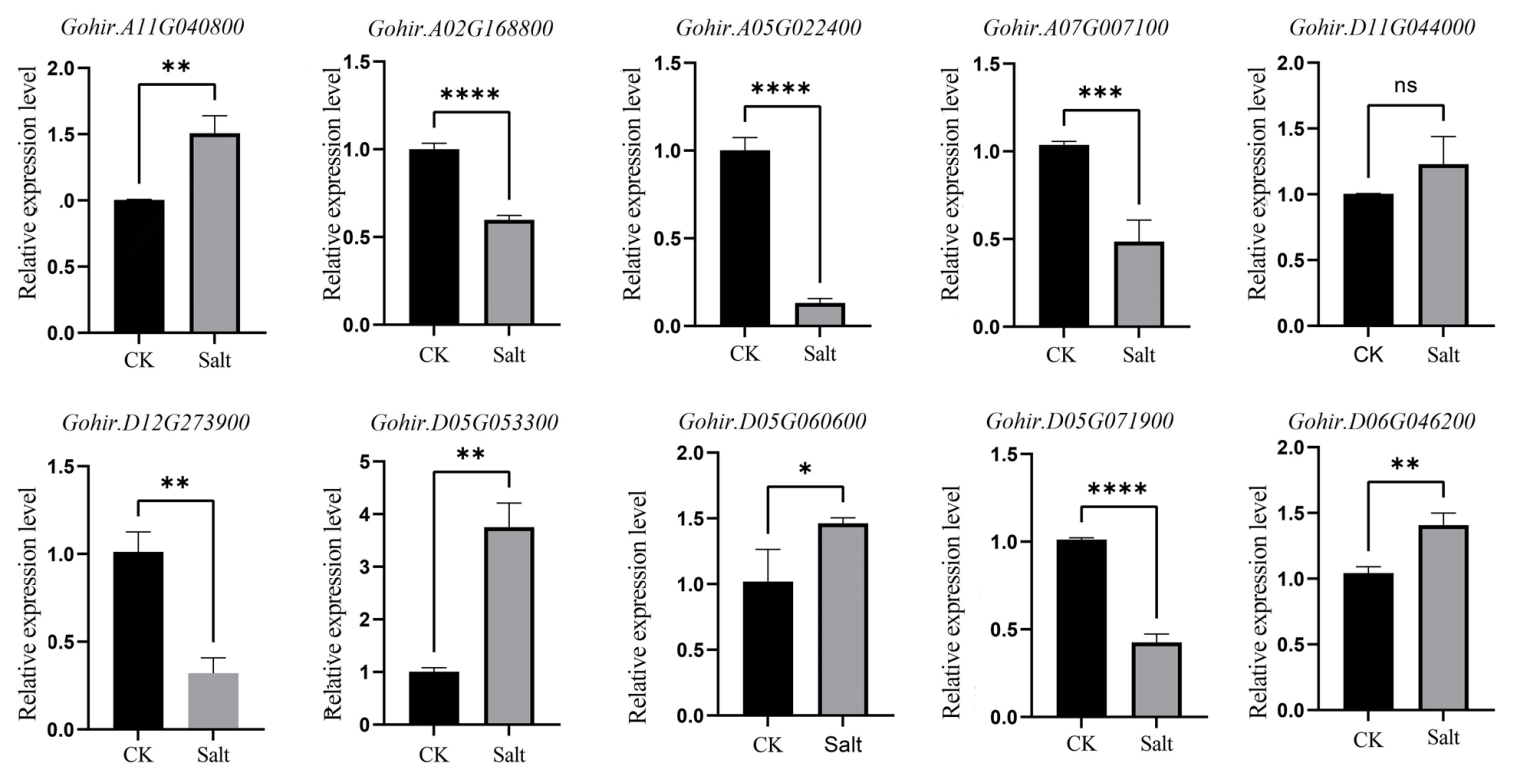
RT‒qPCR analysis of ALDH family in *Gossypium hirsutum* under salt treatment. The symbols *, **, *** and ****, represent significant differences of *P* < 0.05, *P* < 0.01, *P* < 0.001 and *P* < 0.0001, respectively, and T-test was used to compare the expression differences of ALDH gene family between control and salt treatment

The identification of *cis*-acting elements associated with the abiotic stress response in the promoter region of the ALDH gene suggested that these elements may be involved in different abiotic stress response pathways. At the same time, gene expression patterns can provide important clues to gene function. Therefore, to further confirm the molecular function of the ALDH genes in response to abiotic stress, we randomly selected ten genes in *G. hirsutum* and analyzed the expression levels of these 10 genes in leaves after salt treatment for 24 h. Except for the expression level of *Gohir. D11G044000* was not significant, the expression levels of *Gohir. A11G040800*, *Gohir. D05G053300*, *Gohir. D06G0046200* and *Gohir. D05G060600* increased significantly under salt stress, while the expression levels of *Gohir.A02G168800*, *Gohir.A05G022400*, *Gohir.A07G007100*, *Gohir.D12G273900* and *Gohir.D05G071900* decreased significantly (Fig. 7), indicating that these genes may play a role in the response to salt stress. In conclusion, these results suggest that the ALDH gene family is indeed involved in the salt stress response, but the response mechanisms of different genes to salt stress are different.

### Virus-induced gene silencing (VIGS) of ALDH genes leads to increased sensitivity of plants to salt stress

**Fig. 8:**
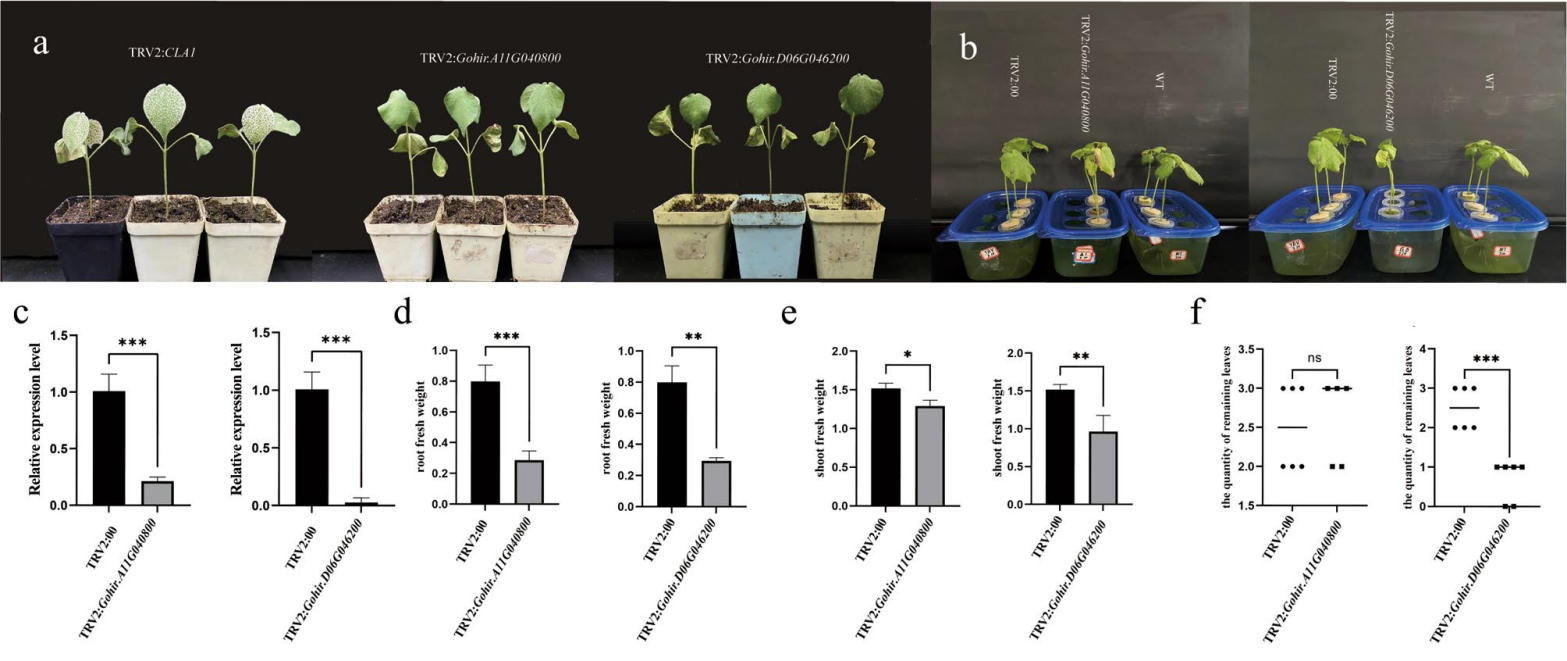
Virus-induced gene silencing of two ALDH genes in *Gossypium hirsutum*. a: albinism phenotype on day 14 of Agrobacterium infection, left: positive control, middle: TRV2:*Gohir.A11G040800*, right: TRV2:*Gohir.D06G046200*. b: Negative controls, TRV2:*Gohir.A11G040800* and TRV2:*Gohir.D06G046200* were transferred to Hoagland Nutrient solution and treated with 300 mmol salt water for 10 days. c: Relative gene expression in silenced plants versus negative control. d: root fresh weight of silenced and negative control plants. e: shoot fresh weight of silenced and negative control plants. f: Remaining leaves of silenced versus negative control plants. Note: Error bars are means of three replicates ± SD; T-test was used for significance test

VIGS is an effective way to study gene function. To investigate the role of ALDH family members in the growth and development of cotton, we constructed a VIGS vector to silence *Gohir.A11G040800* and *Gohir.D06G046200* in a salt-tolerant *G. hirsutum* variety “Xiang FZ031”. As shown in Fig. 2, *Gohir.A11G040800* and *Gohir.D06G046200* are located at the top of the chromosome. Genes located at the chromosome top may be protected by the telomere, thus maintaining relative stability during the genetic process [17]. Additionally, as shown in Fig. 3, the motif distribution and intron-exon structure of *Gohir.A11G040800* and *Gohir.D06G046200* are highly conserved. This may suggest that these two genes play significant roles in evolution. In VIGS experiment, we chose the CLA as the positive control and the TRV as the negative control. *GhCLA*(cloroplastos alterados) is a homologous gene to *AtCLA1*, which is responsible for chloroplast development, and its mutant (*cla1*) has an albino phenotype [18]. TRV belongs to the genus Tobravirus (family Virgaviridae), and the TRV vector has been shown to be useful in *G. hirsutum* and *G. barbadense* by silencing the chloroplastos alterados 1 (*CLA1*) gene [19, 20], so we chose TRV2:00 as the negative control and *CLA1* as the positive control. Approximately 2 weeks after *Agrobacterium* infection, the true leaves of TRV2:*CLA1* developed an albino phenotype (Fig. 8a), indicating that the VIGS procedure is correct and effective. Afterward, except for the positive control plants, we transferred the other plants to Hoagland nutrient solution and treated them with 300 mmol salt solution for 10 days. Compared with wild type (WT) and TRV2:00, the leaves of TRV2:*Gohir.A11G040800* wilted more seriously. TRV2:*Gohir.D06G046200* showed defoliation phenomena (Fig. 8b&c). The number of remaining leaves, root fresh weight and shoot fresh weight were then further measured. Compared with the control group, the root fresh weight and shoot fresh weight of silent plants were significantly reduced (Fig. 8d), and the residual leaf weight of TRV2:*Gohir.D06G046200* was significantly reduced (Fig. 8e). The second true leaf from TRV2:*Gohir.A11G040800*, TRV2:*Gohir.D06G046200*, and the related negative control were collected to extract RNA for RT‒qPCR to verify the effect of gene silencing. The expression levels of these genes in TRV2:*Gohir.A11G040800* and TRV2:*Gohir.D06G046200* were significantly inhibited when the silenced plants were compared with unloaded plants, which demonstrated the success of gene silencing (Fig. 8d&f). In conclusion, VIGS experiments demonstrated that ALDH gene family members reduced their tolerance to salt stress after silencing, suggesting that *Gohir.A11G040800* and *Gohir.D06G046200* may be involved in cotton’s response to salt stress.

## Discussion

A gene family is a group of genes derived from the same ancestor, producing two or more copies of the same gene through gene replication [21]. Aldehyde dehydrogenases (ALDHs) act as “aldehyde scavenger” in plants, eliminating active aldehydes, and thus play a crucial role in responding to stress [22]. This gene family includes NAD or NADP-dependent enzymes, which play an important role in reducing the toxic effects of aldehydes by converting them into corresponding carboxylic acids [23]. At present, the ALDH family has been extensively studied in a variety of plants, including mosses and algae [24], tomatoes [25]and apples [26], et al. However, not much information is currently available about the ALDH gene family in cotton. However, there are also some related studies on the role of ALDH gene family in cotton under abiotic stress, such as GUO et al. analyzed the gene expression of this gene family under high drought and high salt conditions through bioinformatics and RT‒qPCR in *Gossypium hirsutum*, *Gossypium arboreum* and *Gossypium barbadense* [11], and Dong et al. also studied ALDH gene family under salt stress in *Gossypium hirsutum*, *Gossypium arboreum* and *Gossypium raimondii*. RT‒qPCR was also used to analyze the expression levels of this gene family under different intensities of salt stress [13]. Cotton is one of the most important natural fiber crops, which also provides edible oil and biofuel [27]. With the completion of cotton genome sequencing and the development of plant genetics, we can systematically study the structure, location and function of cotton ALDH gene family. It provides basic biological information for further study on the function of cotton ALDH genes.

In this study, genomic files of *G. hirsutum*, *G. raimondii*, and *G. arboreum* were downloaded from the Phytozome and CottonFGD databases, among which allotetraploid *G. hirsutum* was evolved from genomic hybridization and subsequent polyploid of diploid cotton [28]. A total of 114 ALDH genes were identified in the three cotton species. Phylogenetic analysis showed that ALDH members could be divided into six subgroups, and gene structure and motif composition analysis showed that most ALDHs in the same subgroup had similar gene structure and motif distribution. In the early stages of gene amplification, some genes lose introns over time [29], and genes without introns may evolve rapidly when introns are under selection pressure, while genes with larger or more introns are more likely to evolve [30]. Gene replication events play an important role in plant genome variation, which leads to the generation of new genes and genetic regulatory pathways. Gene replication (including tandem gene replication and fragment gene replication) is the main driver of gene family expansion [31]. Our analysis showed that there was neither a large increase nor a large decrease in genes from the ALDH gene family in *G. hirsutum*, and it is speculated that some ALDH genes in cotton gradually lost introns and gained functional evolution over time. The results of Ka/Ks and collinear analysis were similar. The collinearity of ALDH in tetraploid *G. hirsutum* was mainly concentrated among homologous chromosomes, and most of the genes had undergone evolutionary selection during evolution. That is, base substitutions on the coding sequences of these genes do not change the composition of the proteins, so they are not affected by natural selection, and the function of these genes is largely preserved [32].

Stress response regulates the expression of specific genes at the transcriptional level through transcription factors that bind to specific *cis*-elements [33]. Consequently, in order to further understand the possible role of ALDHs in cotton under different environmental stresses, we analyzed the distribution of *cis*-elements in the 1400 bp regions upstream of ALDHs promoters. Many *cis*-elements related to plant abiotic stress response have been found. For example, ABRE has been shown to respond to osmotic stress in plants [34], suggesting that ABRE may be involved in cotton’s regulation of abiotic stress. To better understand the role of ALDHs in cotton abiotic stress, we studied the expression of ALDHs under normal conditions and salt stress. Ten of the 57 ALDH members in *G. hirsutum* were randomly selected for RT‒qPCR to explore their expression levels. The results showed that the expression levels of 4 genes increased significantly under salt stress whereas 5 genes decreased significantly. These genes are involved in the response to salt stress in *G. hirsutum*. Two genes with significantly increased expression levels, *Gohir.A11G040800* and *Gohir.D06G046200*, were silenced by VIGS in a salt-tolerant *G. hirsutum* variety XiangFZ031. The results showed that after *Gohir.A11G040800* was silenced, The leaves showed obvious wilting phenomenon, and after *Gohir.D06G046200* was silenced, the leaves showed obvious shedding phenomenon, and the root and shoot fresh weight also decreased significantly, indicating that the silencing of these two genes negatively affected the tolerance to salt stress.

In conclusion, our study demonstrates that the ALDH gene family is indeed involved in the response to salt stress in cotton.

## Conclusions

Cotton is a pioneer crop in saline-alkali land and is also an important fiber and oil crop. A genome-wide study, RT-qPCR profiling and gene silencing were performed to characterize aldehyde dehydrogenases (ALDHs) genes and their role in the response to cotton to salt stress. In total, 114 genes were identified in three Gossypium species, *G. arboreum*, *G. raimondii*, *G. hirsutum*. Gene silencing of *Gohir.A11G040800* from *G.hirsutum* makes the leaves to wither and scorch, and gene silencing of *Gohir.D06G046200* from *G. hirsutum* makes the leaves to fall off. Taken together, these results suggest that the ALDH gene family is indeed involved in the salt stress response of *G. hirsutum*.

## Materials and methods

### Plant materials

In this study, a *Gossypium hirsutum* variety CRI 12 was used for RT‒qPCR (*Gossypium hirsutum* zhong12). CRI12 cotton plants were grown in an artificial climate chamber with a temperature of 25℃and a 16 h/8 h light to dark ratio. After the first true leaf appeared, the cotton plants were watered with 350 mmol of salt water as salt treatment and fresh water as control, and RNA was extrected with the first true leaf 24 h later. A salt-tolerant *G. hirsutum* variety XiangFZ031 was used for VIGS. Cotton plants were also grown in an artificial climate chamber with a temperature of 25℃and a 16 h/8 h light to dark ratio. When the albino phenotype appeared, it was transferred to Hoagland nutrient solution and treated with 350 mmol of salt water for 10 days.

### Identification and evolutionary analysis of ALDH gene family members in cotton

The genome data, including CDS, gene annotation files, and protein sequence files of *G. hirsutum* (V3.1) and *G. raimondii* (V2.1) were downloaded from the Phytozome V13 database (https://phytozome-next.jgi.doe.gov/) [35–37]. The genome and protein sequence of *G. arboreum* were obtained from the CottonFGD genome database (https://cottonfgd.net/) [38], and the CDSs of *G. arboreum* were extracted through TBtools using gff files and fasta files. The Pfam database (http://pfam-legacy.xfam.org/) was used to search for the Pfam number (PF00171) of the ALDH gene family and download the Hidden Markov model (HMM) of the ALDH gene family [39]. HMMER3.0 software and the BLASTP comparison program were used to search for sequences containing ALDH protein domains. The E value is set to E-20 for screening candidate protein sequences. Candidate protein sequences were uploaded into SMART database (http://smart.embl.de/) [40], Pfam database (http://pfam-legacy.xfam.org/) and NCBI database for CDD search (https://www.ncbi.nlm.nih.gov/cdd/) for reidentification [41]. The amino acid sequences of all members of ALDH gene family in the three cotton species were analyzed by ExPASy software (http://www.ExPASy.org) [42], and the amino acid length and isoelectric point (PI) were calculated. To further analyze the evolutionary relationship of ALDH gene family, MEGA7 software was applied to construct the phylogenetic tree by neighbor-joining method after obtaining the sequence through multiple sequence comparison.

### Evolutionary selection pressure analysis of ALDH gene family

We constructed a local index for *G. hirsutum* genomic gene sequence, and compared the whole CDS data with BLASTP through Blastall program, with a E value of e^-20^ to obtain *G. hirsutum* genome comparison results. The synonymous substitution rates (Ks) and nonsynonymous substitution rates (Ka) of ALDH genes in cotton were calculated using Calculator 2.0 to analyze the selection pressure on the genes during evolution.

### Chromosome localization analysis of ALDH gene family

ALDH gene location information on the chromosome was obtained from three different cotton gene annotation files, and then TBtools was used to map genes’ location on chromosomes.

### Analysis of ALDH family gene structure and conserved motifs of three cotton species

MEME website (http://memesuite.org/) was used to analyze the conservative motifs of ALDH family members [43]. The parameter is set to search a total of 10 motifs, with the shortest motif length being 6 base pairs and the longest motif length being 50 base pairs. To analyze the structure information of ALDH genes, exons, CDS, 3’UTR and 5’UTR position information of ALDH genes on chromosome were extracted. The structural information of the ALDH gene family was then analyzed and the gene structure was mapped using the online website of GSDS. Finally, TBtools software was used to integrate and visualize images of ALDH phylogenetic trees, gene structures and conserved motifs.

### *Cis*-acting element analysis

To explore the related functions of gene expression regulation, the 1400 bp promoter sequences upstream of the promoters were obtained from the *G. hirsutum* genome file and the *cis*-acting elements of the genes were analyzed. The PlantCARE database (http://bioinformatics.psb.ugent.be/webtools/plantcare/html/) was used to identify and analyze *cis* elements of these genes [44], and then the GSDS online website (http://gsds.gao-lab.org/) was used to draw the figure.

### Collinearity analysis of ALDH gene family

By comparing the sequences of all ALDH proteins, MCScanX and circos software were used to determine and analyze the repeatability and homology of ALDH proteins in the cotton genome.

### RT‒qPCR analysis

RNA was extracted using a special RNA extraction kit for polysaccharide polyphenols (Tiangen) and reverse-transcribed using the HiScript III RT SuperMix for qPCR (+ gDNA Wiper) kit (Vazyme, Nanjing, China). Real-time quantitative PCR analysis was performed using ChamQ SYBR qPCR Master Mix (LowROX Premixed) kit (Vazyme, Nanjing, China). Primers of ALDH gene family were designed using Primer Premier 6.0 and listed in Supplementary Table S4(Table S4). The reaction volume was 20 µL, and the amplification procedures were as follows: predenaturation at 95℃ for 30 s, denaturation at 95℃ for 10 s, and 40 cycles of annealing at 60℃ for 30 s. Each gene is repeated for three biological replicates and three technically replicates. Actin was used as the reference gene, and the expression level of related genes was quantified by 2^^-ΔΔCt^.

### Virus-induced gene silencing (VIGS) experiment

A *G. hirsutum* varitey XiangFZ031 was used for VIGS experiment. Cotton seeds were soaked in carbendazim, sterilized, and then sowed in pots filled with nutrient soil and vermiculite (3:1 ratio). The greenhouse temperature is maintained at 25°C with a 16 h/8 h of light to dark ratio. The VIGS experiment was performed when the cotyledon of cotton was fully unfolded and the first true leaf had just appeared. Primers designed with Prime 6 for VIGS were ligated to pTRV2 vectors to obtain recombinant expression vectors. *Gohir.A11G040800* is transformed by an upstream primer of 5’-GTGCCATTGCTGCTAAGA-3’ and a downstream primer of 5’-ACAGATGCCAACTCAGAAG-3’. *Gohir.D06G046200* was transformed by an upstream primer of 5’-GAATGGAGAAGGACTCAGTT-3’ and a downstream primer of 5’-GGATCAAGAGACAGCAAGA-3’. The primer sequences were inserted into the TRV vector and treated with *Sac* I and *Xba* I enzymes to construct TRV2: *Gohir.A11G040800* and TRV2: *Gohir.D06G046200*. The plasmid is then transformed into *Agrobacterium tumefaciens* (GV3101). After the positive clones were screened out, the bacterial solution was injected into the cotyledon of cotton seedlings using sterile syringes. After 48 h of dark treatment, it was transferred to an artificial climate chamber. The positive control showed albino phenotype about two weeks later. The negative control plants and silent plants were then transferred to Hoagland nutrient solution and treated with 350 mmol brine for 10 days. The root fresh weight, shoot fresh weight and the number of remaining leaves of these plants were measured, respectively.

### Electronic supplementary material

Below is the link to the electronic supplementary material.

Supplementary Material 1

Supplementary Material 2

Supplementary Material 3

Supplementary Material 4

## Data Availability

Data is provided within the manuscript or supplementary information files.

## Abbreviations

ALDH: Aldehyde dehydrogenase
RT-qPCR: Quantitative real-time polymerase chain reaction
GSDS: Gene Structure Display Server
MW: Molecular weight
PI: Theoretical isoelectric point
HMM: Hidden Markov model
MCScanX: Multiple Collinearity Scan toolkit
